# Distributed Learning in the Classroom: Effects of Rereading Schedules Depend on Time of Test

**DOI:** 10.3389/fpsyg.2018.02517

**Published:** 2019-01-09

**Authors:** Carla E. Greving, Tobias Richter

**Affiliations:** ^1^Department of Cognitive Psychology, University of Kassel, Kassel, Germany; ^2^Department of Psychology IV – Educational Psychology, University of Würzburg, Würzburg, Germany

**Keywords:** distributed learning, spacing effect, lag effect, retention interval, rereading

## Abstract

Research with adults in laboratory settings has shown that distributed rereading is a beneficial learning strategy but its effects depend on time of test. When learning outcomes are measured immediately after rereading, distributed rereading yields no benefits or even detrimental effects on learning, but the beneficial effects emerge two days later. In a preregistered experiment, the effects of distributed rereading were investigated in a classroom setting with school students. Seventh-graders (*N* = 191) reread a text either immediately or after 1 week. Learning outcomes were measured after 4 min or 1 week. Participants in the distributed rereading condition reread the text more slowly, predicted their learning success to be lower, and reported a lower on-task focus. At the shorter retention interval, massed rereading outperformed distributed rereading in terms of learning outcomes. Contrary to students in the massed condition, students in the distributed condition showed no forgetting from the short to the long retention interval. As a result, they performed equally well as the students in the massed condition at the longer retention interval. Our results indicate that distributed rereading makes learning more demanding and difficult and leads to higher effort during rereading. Its effects on learning depend on time of test, but no beneficial effects were found, not even at the delayed test.

## Introduction

Learning from text is essential for learning in school and academic settings. But how should we read to foster long-term learning? Distributing learning episodes of study material over a longer time instead of cramming in one session has shown to be a beneficial learning strategy, especially for longer retention intervals (spacing effect; Cepeda et al., 2006). Given that distributed learning is usually perceived as more difficult by the learners than massed learning, distributed learning may be regarded as a desirable learning difficulty (Bjork and Bjork, 2011). The assumption that distributed learning benefits long-term learning seems to hold also for learning with texts. Research with adults in laboratory settings has repeatedly shown that distributed rereading of a text is more effective for long-term retention than massed rereading (Glover and Corkill, 1987; Krug et al., 1990; Rawson and Kintsch, 2005; Verkoeijen et al., 2008; Rawson, 2012). However, the effect of distributed vs. massed rereading has not yet been investigated with younger learners in real-world educational settings. In a preregistered experiment (Greving and Richter, 2017), we investigated distributed rereading in a school environment with seventh-graders. In this article, we first discuss desirable difficulties and distributed learning in general. We also provide an overview of empirical findings on the effects of distributed rereading and then introduce the current experiment.

### Distributed Learning and Desirable Difficulties

Distributed learning is one of several learning strategies labeled as desirable difficulties (Bjork, 1994; Bjork and Bjork, 2011; Lipowsky et al., 2015). These learning strategies share two key features. They seem to make learning more difficult during learning, but they enhance learning outcomes in the long term. One factor assumed to make learning more difficult but foster long-term retention is the time between repetitions of learning material.

Distributed learning refers to learning schedules in which repetitions of the information to be learned (e.g., a new word in a foreign language) is distributed over several (at least two) learning sessions instead of learning in only one session. For example, when using flashcards to learn vocabulary of a new language, the inter-study interval should be increased between the repetitions of the same flashcard. The term distributed learning encompasses the spacing and the lag effect. The spacing effect refers to the finding that any inter-study interval leads to better learning than massed learning (i.e., learning with an inter-study interval of zero). However, in studies investigating the lag effect, learning outcomes are compared between learning schedules with different inter-study intervals.

The spacing effect, as defined by Cepeda et al. (2006), is a robust effect that is not moderated by the retention interval. That is, distributed learning is usually better than massed learning. In contrast, the lag effect designates a non-monotonic effect of the inter-study interval. Learning performance increases with longer inter-study intervals until the effect reaches a peak, after which the performance decreases with even longer inter-study intervals. Moreover, the lag effect depends on the retention interval. Learning over longer retention intervals seems to benefit from longer inter-study intervals (Glenberg, 1976; Cepeda et al., 2006, 2008). Different processes might account for the spacing and the lag effect (Cepeda et al., 2006; Küpper-Tetzel and Erdfelder, 2012). Retrieval processes during retention tests have been discussed as explanations for the spacing effect, whereas the lag effect may be explained by different encoding strategies (e.g., retrieval of the first encoding of an item) during learning or maintenance after learning. One key mechanism might be the retrieval of stored information from the first learning occasion during the second learning occasion. Study-phase retrieval theories suggest that successful retrieval of the first learning occasion is needed to strengthen the memory trace and thus prevent forgetting (Thios and D’Agostino, 1976; Cepeda et al., 2008; Delaney et al., 2010).

### Distributed Rereading as Desirable Difficulty in Learning

The long-term benefits of distributed learning have been shown for a wide range of materials, from simple motoric tasks (e.g., Baddeley and Longman, 1978) and simple materials such as vocabulary (e.g., Kornell, 2009) to complex learning materials such as texts (Rawson and Kintsch, 2005). Rereading texts clearly seems to be a common learning strategy widely used by students (e.g., Karpicke et al., 2009; Gagnon and Cormier, 2018). Contrary to common sense, rereading a text immediately after the first reading often provides at best marginal gains in the learning outcome compared to reading the text only once (Callender and McDaniel, 2009). However, rereading the text in a distributed fashion might be a better strategy (Glover and Corkill, 1987; Krug et al., 1990; Verkoeijen et al., 2008), but its effectiveness seems to depend on the retention interval (Gordon, 1925; Rawson and Kintsch, 2005; Rawson, 2012).

Rawson and Kintsch (2005, Experiment 1) investigated the rereading and retention interval effects by comparing recall and text comprehension performance of undergraduates who read an expository text about carbon sequestration (1730 words) once or twice either immediately after the first reading or 1 week later. Recall and text comprehension performance were measured either immediately after reading or after a delay of 2 days. When learning outcomes were assessed immediately after reading, students who had read the text twice in the massed condition outperformed students in the single reading condition in recall and text comprehension performance, whereas no differences were found between the distributed reading and single reading conditions. Thus, at the short retention interval, no benefit of distributed rereading was found. In the recall performance, students in the massed condition even outperformed students in the distributed condition. But when learning outcomes were measured 2 days later, a different pattern emerged. Students in the distributed condition outperformed those in the massed and single reading condition in recall and comprehension performance. Thus, the benefits of distributed rereading depended on time of test.

Rawson (2012; also see Rawson and Kintsch, 2005, Experiment 2) replicated the interaction between the rereading and retention intervals in three experiments with undergraduates and a text about the portrayal of historical events in Hollywood films (1541 words in length). In all experiments, they found no difference between the rereading conditions at the short retention interval, whereas students in the distributed condition outperformed students in the condition with immediate rereading at the long retention interval. In addition, Rawson and Kintsch (2005) as well as Rawson (2012) measured the reading times and found a decrease in reading time between the first reading and the rereading. The decrease was greater for the group with immediate rereading. Thus, participants in the distributed condition spent more time reading the second text than participants in the massed condition.

In sum, the interaction between rereading schedules and retention intervals on testing performance seems to be robust in college students in laboratory settings. The differences in reading times suggest that readers spend greater cognitive effort in distributed vs. massed rereading.

### Meta-Cognitive Judgments of the Learning Process and Distributed Learning

Although distributed learning is an effective learning strategy, students seem to underrate the effectiveness of distributing their learning time in their metacognitive judgments of the learning process (for a review see Son and Simon, 2012). One core type of meta-cognitive judgments of the learning process, which is often assessed immediately after learning, is the estimated proportion of correctly recalled items. These judgments are influenced by many cues, as for example the perceived difficulty of a to-be-learned item (Koriat, 1997; see also Vössing et al., 2017, for the influence of difficulty on the accuracy of those judgments). For example, Kornell (2009) investigated distributed vs. massed learning of vocabulary with flashcards. Despite the objective advantage of a distributed learning strategy, participants estimated a higher percentage of correct recalled items of the massed learned items than of the distributed learned items. A possible explanation for this pattern is the lower experienced fluency during distributed learning (Alter and Oppenheimer, 2009; Bjork et al., 2013). As distributed learning should induce a (desirable) difficulty, learners might also perceive learning as more difficult when the materials are presented in a distributed instead of a massed fashion. Thus, distributed rereading might not only affect the learning outcome and the reading time, it might also alter the meta-cognitive judgments of the learning process. However, to our knowledge, the effects of distributed rereading on the meta-cognitive judgments of the learning process have not yet been investigated. As texts are more complex learning materials than single words, the question arises whether distributed rereading also induces a perceivable difficulty and if so, whether meta-cognitive judgments of learning are affected by the difficulty induced by distributed rereading.

### Distributed Learning in Real-World Educational Settings

The effects of distributed learning are well investigated in laboratory settings but only few studies have been conducted that examine distributed learning in real-world educational settings (Küpper-Tetzel, 2014). However, to give recommendations to teachers to apply distributed learning, studies are needed to investigate whether this teaching strategy is indeed beneficial in real-world educational settings. Such settings differ in a number of respects from laboratory settings. For example, distributed learning occurs embedded in other instructional activities, learning usually is usually more self-regulated and is based on more complex materials.

Furthermore, the studies introduced above have been conducted with adult learners, especially with undergraduates. Experimental settings in school and with younger learners might confront researchers with more heterogeneous samples. Whereas undergraduate university students often represent a highly selected group of learners on a relatively high level of ability, in a secondary school setting, high-capacity students often visit the same class as low-capacity learners. Interestingly, advantages of distributed learning were shown for vocabulary learning with school students in classroom settings (Bloom and Shuell, 1981; Sobel et al., 2011; Küpper-Tetzel et al., 2014), and distributed learning of scientific concepts and laws seems to foster long-term learning (Grote, 1995; Vlach and Sandhofer, 2012; Gluckman et al., 2014; Vlach, 2014; Kapler et al., 2015). However, in an experiment conducted by Goossens et al. (2016), a longer lag failed to facilitate primary school vocabulary learning in a classroom learning scenario compared to a shorter lag condition.

Additionally, learning abilities (for example skill learning, Schiff and Vakil, 2015), general cognitive prerequisites for learning such as working memory capacity (Gathercole et al., 2004) and reading comprehension skills (Perfetti et al., 2005) that are especially important for learning from text underlie huge developmental changes. Thus, as matter of principle, a learning method that has been shown to be beneficial for adult learners is not guaranteed to work for younger learners. However, some studies suggest that distributed learning seems to be as beneficial for young children as for young adults (Toppino et al., 1991; Seabrook et al., 2005).

To summarize, despite the contrary findings regarding the lag effect of Goossens et al. (2016), distributed learning promises to be a beneficial learning strategy even for school-aged learners and in real-world educational settings. However, distributed rereading of expository texts has not yet been investigated with younger learners and it is unclear whether the findings for adult learners generalize to this population.

### The Role of Prior Knowledge in Distributed Rereading

Prior knowledge is arguably the most important learner characteristic for learning from text (e.g., Kintsch, 1998), even more important than verbal abilities (Schneider et al., 1989). Moreover, prior knowledge has been shown to moderate the effects of text difficulty on learning from texts. McNamara and Kintsch (1996) demonstrated that the comprehension of junior high school students with low prior knowledge benefited from more coherent and thus easier texts, whereas the comprehension of students with higher prior knowledge benefit from less coherent and thus more difficult texts. As distributed rereading should also lead to higher difficulty in rereading, the question arises whether distributed rereading is also only beneficial for students with high(er) prior knowledge. In their experiment with university students, Rawson and Kintsch (2005) measured prior knowledge but did not find an interaction with the rereading schedule. Still, prior knowledge might play a role for distributed rereading in a school context, where the distribution of prior knowledge is likely to differ from the distribution typically found at universities.

### The Current Experiment

In this preregistered experiment (Greving and Richter, 2017), we investigated the effects of massed and distributed rereading on short- and long-term retention with seventh-graders in the classroom. In addition to reading times, metacognitive judgments were obtained to gain insights into the learning process.

Based on the experimental design of Rawson and Kintsch (2005), participants twice read curriculum-orientated texts about the bacterial cell. The rereading occurred either immediately after the first reading or 1 week later. Recall and text comprehension performance were measured 5 min after rereading (short retention interval) or 1 week later (long retention interval). Thus, the present experiment is the first to investigate the effects of distributed rereading on the learning outcomes of school students but to also concurrently expand the research on the effects on metacognitive processes.

Following the findings of Rawson and Kintsch (2005), we expected that distributed rereading would have beneficial effects on learning in recall and text comprehension performance. However, the expected beneficial effect of distributed rereading was expected to depend on time of test. No differences were expected at the short retention interval, whereas the benefits of distributed rereading was expected to be significant at the longer retention interval (Hypothesis 1). In addition, we expected that because of forgetting, the learning outcome should decrease between the retention intervals (Hypothesis 2). Considering the significant influence of prior knowledge on learning with texts, we also estimated the effects of domain-specific prior knowledge. We first assumed that students would learn more from the texts the higher their prior knowledge (Hypothesis 3). This hypothesis is backed up by a large body of research demonstrating the importance of prior knowledge in learning from text (e.g., Schneider et al., 1989). Although the hypothesis is not novel, testing it in the present experiment is important to ensure that students indeed used their prior knowledge to understand and learn from the text. Furthermore, we addressed the exploratory research question whether the effects of distributed rereading would depend on prior knowledge (similar to other measures that make text comprehension more difficult, such as low-coherence texts, McNamara et al., 1996).

We also hypothesized that distributed rereading would lead to greater cognitive effort and hence longer reading times in the second text presentation (Hypothesis 4). Regarding metacognitive judgments of learning, we expected that students would perceive distributed rereading as more difficult (Hypothesis 5) and rate the learning process as less successful (Hypothesis 6). Despite the perceived disadvantage, we expected that students would be more focused on the task (Hypothesis 7) during distributed rereading.

## Materials and Methods

### Participants

The sample included 191 (53% female) seventh-grade students from eight classes and three different schools (German Gymnasium and comprehensive schools). The average age of participating students was 12.94 years (*SD* = 0.39). Students participated only if their parents had given their permission (97% permission; students without permission took quizzes during sessions). Students were randomly assigned to the four experimental learning conditions: massed learning condition with delayed measurement (*n* = 49), massed learning with immediate measurement (*n* = 47), distributed learning with delayed measurement (*n* = 48), and distributed learning with immediate measurement (*n* = 47). As a reward, the students received sweets after each session and a magic cube puzzle after the last session.

Twenty students missed at least one of the learning sessions, thus did not read the texts twice. Therefore, their data were excluded from all analyses. This participant loss resulted in the following group sizes: massed/delayed (*n* = 47), massed/immediate (*n* = 47), distributed/delayed (*n* = 37), and distributed/immediate (*n* = 40). Additionally, 26 students missed the test or the assessment of prior knowledge, resulting in the following group sizes in the analysis of free recall and text comprehension performance: massed/delayed (*n* = 36), massed/immediate (*n* = 45), distributed/delayed (*n* = 26), and distributed/immediate (*n* = 38).

### Text Materials

The experimental text was an expository text about the bacterial cell (length 74 sentences, 977 words). The bacterial cell structure is part of the extended curriculum of biology science classes in the State of Hessen (Germany) where the study was conducted. However, the bacterial cell structure is usually not covered in class because it is too small to be microscopable in school contexts. Thus, it was unlikely that the students had prior knowledge about the bacterial cell itself, but they might have had prior knowledge about cells in general. A complementary image illustrating the structure of the cell that was also explained in the text was presented adjacent to the text. The image was presented stable, thus the reader could always integrate text and image. This is comparable to the typical layout of text books of biology, in which the information about the respective cell is mostly accompanied by illustrations of its structures. The text had a Flesh reading ease score of 54 (German formula, Amstad, 1978).

### Assessment of Learner Characteristics

Participants’ first language and diagnosed reading and writing disability were reported by their teachers in a teacher questionnaire. Moreover, further learner characteristics were assessed via standardized tests. Besides the domain-specific prior knowledge, we assessed reading ability, working memory capacity, and knowledge about reading strategies as further abilities which are associated with reading and learning skills and thus can be seen as prerequisites for learning (see “Distributed Learning in Real-World Educational Settings”). A randomized block design was used to ensure that the experimental groups are matched with respect to these abilities.

#### Domain-Specific Prior Knowledge

Participants were asked to answer five open-ended questions and to label the components of the plant cell and the bacterial cell in a schematic image. The questions covered knowledge related to the bacterial cell (e.g., function of cells, knowledge about genetic information), but were asked in a way to promote the students to write up any prior knowledge. For example, one question was “What is a plant or animal cell? Please write down everything you know about those cells.” The questions have been used in two other studies as well (two experimental and one pilot study). In these studies, the scores were highly correlated with the recall performance after reading a preliminary version of the text used in this experiment (pilot study: *r* = 0.72, 95% CI [0.51, 0.84]).The questions were presented in randomized order. Additional to the knowledge questions, we also asked the participants to indicate whether they had encountered the topic before in class or at home. The protocols were scored by two independent raters following a coding scheme. Any answer which was correct even at low level, as for example “something inside an animal,” was given a point, with more points given for more elaborated answers as “An animal or plant cell is a tiny unit of a plant or an animal,” ICC (2,1) = 0.93, 95% CI [0.923, 0.932] (Shrout and Fleiss, 1979).

#### Knowledge About Reading Strategies

Participants completed the Würzburger Lesestrategie-Wissenstest für die Klassen 7-12 (WLST 7-12; Würzburg Reading Strategy Knowledge Test, Schlagmüller and Schneider, 2007; split-half reliability, *r* = 0.90, estimated in a sample of 4490 students in Grades 7-11). The WLST includes six items that require participants to grade the utility of different reading strategies in a given learning situation (on a scale from 1 to 6, corresponding to the German grading system, where 1 is the highest achievement and 6 the lowest).

#### Reading Ability

Participants completed the subtest sentence verification of ELVES, a German-speaking test that assesses the efficiency of basic reading processes at the word and sentence level (Richter and van Holt, 2005). In this task, 16 statements are judged as true or false (verification task). The test score combines reading speed and verification accuracy into an integrated score (Cronbach’s α = 0.58, estimated in the current sample). The reliability of this measure was lower than in previous studies (e.g., Richter and van Holt, 2005 report a Cronbach’s α of 0.87), indicating a relatively high amount of measurement error. However, given that the purpose of the reading ability measure was to match the experimental groups according to this criterion, the reliability of the measure may still be sufficient.

#### Working Memory Capacity

Working memory capacity for text was assessed with a computerized version of the Reading Span Task (RSPAN; Oberauer et al., 2000). The task involves verification judgments for sequentially presented sentences that increase in number throughout the test and the memorization of the final word of each sentence. The test score is the average proportion of correctly recalled words (Cronbach’s α = 0.89, estimated in the current sample).

### Assessment of Learning Outcomes

#### Recall Performance

Recall performance was assessed with a free recall task. Participants were asked to write down as much information that could be recalled from the first part of the text. The participants were given a time limit of 2 min. The free recall protocols were scored by two independent raters, ICC (2,1) = 0.92, 95% CI [0.866, 0.948] (Shrout and Fleiss, 1979).

#### Text Comprehension Performance

Text comprehension performance was assessed with eight short-answer and six single-choice questions (one correct response option and three distractors). For example, one short-answer question was, “A bacteria cell does not have a cell nucleus. But where can you find the genome of the bacteria cell?,” and one single-choice questions was, “To which kind does the bacteria cell belong?,” with the response options (a) Prokaryots, (b) Eukaryots, (c) Plasmid, and (d) Organelle. The additional single-choice questions (compared to Rawson and Kintsch, 2005) were chosen because younger learners in previous (yet unpublished) experiments tended to forego answering the open-ended questions. All questions were literal questions asking for information explicitly stated in the text. The questions had originally been developed for these previous experiments and were optimized for the present experiments regarding item difficulties. The item difficulty (calculated averaged about all learning and retrieval conditions) ranged between 0.01 and 0.72, with a mean difficulty of 0.30 (*SD* = 0.19) in the short-answer questions as well in the single-choice questions (*SD* = 0.10) (corrected for chance success). Answers to the short-answer questions were scored as either incorrect (0) or correct (1) by two independent raters who were blind to the experimental conditions (Cohen’s *κ* = 0.87).

### Assessment of Learning Processes

#### Reading Time

The students read the text in a self-paced fashion with the moving-window method. The text was presented on screen with all sentences blurred except the one the student was currently reading. The students could return to previously read sentences to reread them. Reading times per sentence were assessed and divided by the number of letters in the sentence to account for different sentence lengths.

#### Metacognitive Judgments of the Learning Process

After reading the text for the second time, participants judged the following aspects of the learning/reading process on 5-point Likert scales. They predicted their learning success and rated the perceived reading difficulty. In addition, the perceived on-task focus (three items, one reversed, Cronbach’s α = 0.64, estimated in the current sample) was assessed. Furthermore, they rated the perceived similarity of the two (identical) texts for exploratory purposes. The results for this measure are not reported as they do not contribute to answering the research questions.

### Procedure

All materials were presented on notebook computers with 15.6′′ screens. The experiment was created and presented with the software Inquisit (Inquisit 3, 2011).

**FIGURE 1:**
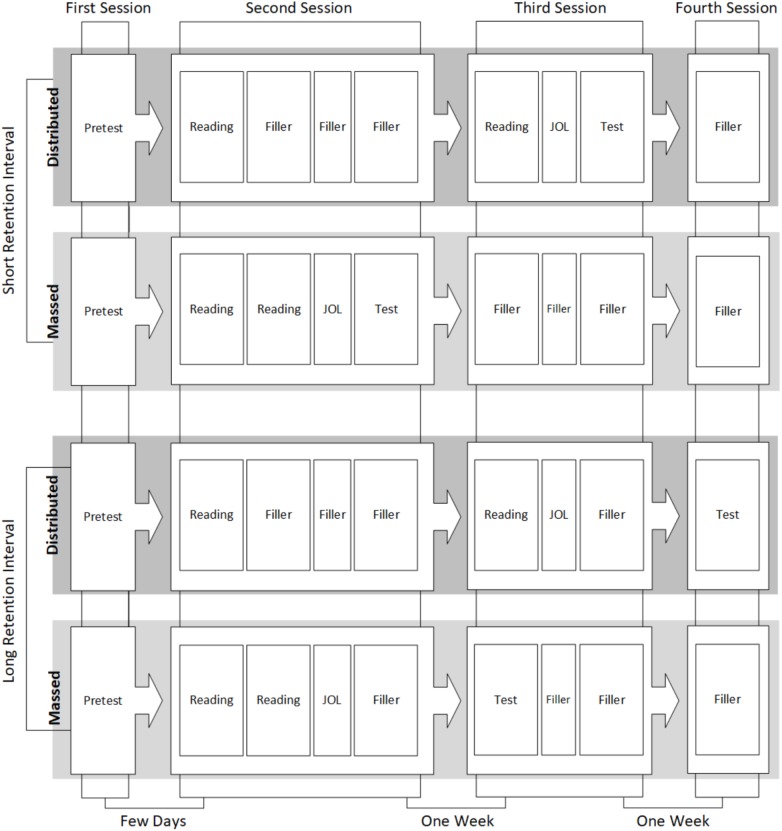
Overview of the experiment procedure. (Reading, reading the text; Filler, filler task; JOL, metacognitive judgments of the learning process; Test, assessment of recall performance and text comprehension performance).

The experiment consisted of four sessions (Figure 1). The pretest took place at the first session, in which the experimental parts were administered collectively in the classroom, supported by instructions on screen. The students completed the prior knowledge test, the WLST, the ELVES, and the RSPAN tests, in this order.

In the further sessions, instructions were given on screen after a short instruction delivered by the experimenter to the whole group.

In the second session, the students either read the experimental text once (distributed) or twice (massed). In the distributed condition, the students received filler tasks after reading. All filler tasks consisted of questions about social media usage and were not analyzed. In the massed condition, the students completed the metacognitive judgments of the learning process. Afterwards, they either were tested (short retention interval) or received a filler task (long retention interval).

In the third session, students read the second text (distributed condition) or received a filler task (massed condition, short retention interval), or the recall test (massed condition, long retention interval). Afterwards, students in the massed condition received a filler task. In the distributed condition, the students completed the metacognitive judgments of the learning process and were then either tested (short retention interval) or received a filler task (long retention interval).

In the fourth session, students were tested (distributed condition, long retention interval) or received a filler task (all other experimental groups).

### Design

We employed a 2 × 2 between-subjects design with matched (parallel) groups and the independent variables learning condition (massed vs. distributed by 1 week) and retention interval (immediate vs. 1 week delayed). To ensure similar capabilities in all learning conditions, we first formed homogeneous blocks of students matched according to first language, reading and writing disabilities, prior knowledge, and reading ability. The students from these groups were then randomly assigned to the experimental conditions. No differences were found between the two learning conditions in working memory capacity, *F*(1,155) = 0.26, *p* = 0.611, and reading ability, *F*(1,155) = 0.41, *p* = 0.521, and between the two groups tested at different retention intervals in working memory, *F*(1,155) = 1.42, *p* = 0.236, and reading ability, *F*(1,155) = 0.08, *p* = 0.777. Likewise, the interaction of the two independent variables was not significant for working memory, *F*(1,155) = 0.01, *p* = 0.940, or reading ability, *F*(1,155) = 0.36, *p* = 0.548. Finally, we found no differences between learning conditions in prior knowledge, *F*(1,155) = 1.69, *p* = 0.195, and reading strategy knowledge, *F*(1,155) = 0.33, *p* = 0.565, or between the two groups tested at different retention intervals in prior knowledge, *F*(1,155) = 0.01, *p* = 0.942, and reading strategy knowledge, *F*(1,155) = 0.01, *p* = 0.930. The interaction was also not significant for prior knowledge, *F*(1,155) = 0.54, *p* = 0.464, or reading strategy knowledge, *F*(1,155) = 2.28, *p* = 0.133.

## Results

We used linear models (recall performance and judgments of learning), linear mixed-effect models (LMM, reading time) and generalized linear mixed-effect models (GLMM, text comprehension performance) with the R packages lme4 (Bates et al., 2015), lmerTest (Kuznetsova et al., 2017) and lsmeans (Lenth, 2016) in the R environment in version 3.4.4 (R Developmental Core Team, 2018). Mixed effect models are the method of choice for analyzing data in educational contexts, which are often characterized by a hierarchical multilevel structure (students nested in classes nested in schools). Moreover, these models are advantageous in experimental contexts when participants and experimental items form a crossed (imperfect) hierarchy (Baayen et al., 2008). We included school, class, student, or item as random effect (random intercept) if the intra-class correlation of the dependent variable exceeded 0.05. Unstandardized regression weights are reported. For interpreting the GLMM results, predicted probabilities (back-transformed from the log odds) for experimental conditions are reported. For all models, the distribution of residuals was inspected visually for normality. All available data points were analyzed; no outliers were excluded. Type 1 error probability was set at 0.05. Directed hypotheses were tested with one-tailed tests.

### Recall Performance

**FIGURE 2:**
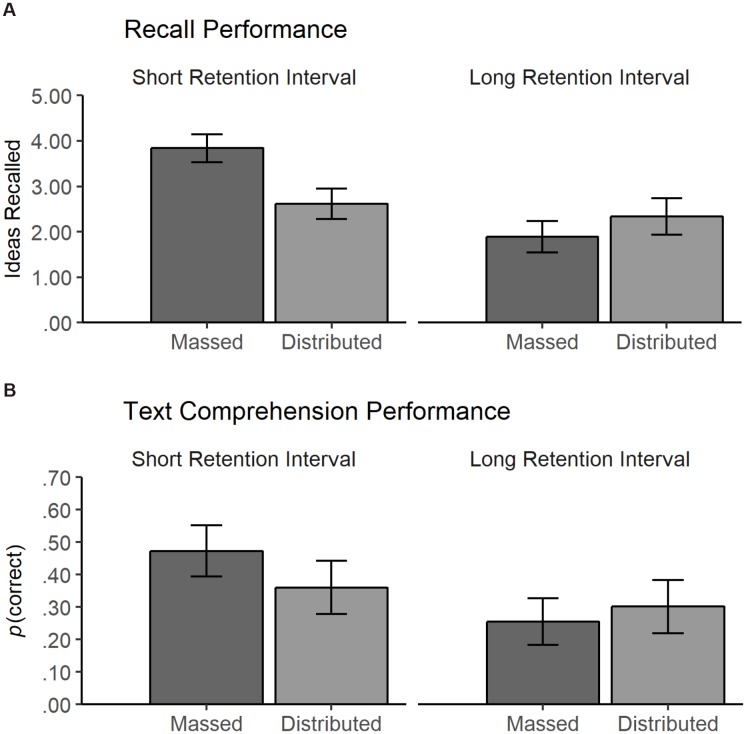
Estimated recall and text comprehension performance in the two learning conditions (massed vs. distributed) at the short and long retention interval; **(A)** mean number of recalled ideas in the free recall task, **(B)** back-transformed probability of a correct answer in the text comprehension test. Error bars represent standard errors.

We estimated a linear model with learning condition (contrast coded: massed = -1, distributed = 1), retention interval (contrast coded: short = -1, long = 1), prior knowledge (z-standardized), and the two- and three-way interactions of these variables as predictors and recall performance as dependent variable. A main effect of retention interval emerged, β = 0.57, *SE* = 0.17, *t*(137) = 3.34, *p* < 0.001, one-tailed, Δ*R^2^* = 0.08. As expected, recall performance was better at the short interval (*M* = 3.21, *SE* = 0.22) than at the long retention interval (*M* = 2.06, *SE* = 0.26). Additionally, students’ recall performance was positively related to their prior knowledge, β = 0.55, *SE* = 0.19, *t*(137) = 2.86, *p* = 0.002, one-tailed, Δ*R^2^* = 0.05. Thus, a difference of one standard deviation in prior knowledge corresponded to a 0.55 difference in the free recall task. No main effect of learning condition was found on recall performance, β = -0.19, *SE* = 0.17, *t*(137) = -1.09, *p* = 0.140, one-tailed, Δ*R^2^* = 0.03, performance in the massed condition (*M* = 2.82, *SE* = 0.22) did not differ from that in the distributed condition (*M* = 2.45, *SE* = 0.26). However, a significant interaction between learning condition and retention interval emerged, β = -0.46, *SE* = 0.17, *t*(137) = -2.687, *p* = 0.004, one-tailed, Δ*R^2^* = 0.04 (Figure 2A). In the massed condition, recall performance decreased from the short (*M* = 3.86, *SE* = 0.30) to the long retention interval (*M* = 1.79, *SE* = 0.34), *t*(137) = -4.60, *p* < 0.001. In contrast, no difference was found between the short interval (*M* = 2.56, *SE* = 0.33) and long retention interval (*M* = 2.34, *SE* = 0.40) in the distributed condition, *t*(137) = -0.43, *p* = 0.666. Conversely, at the shorter retention interval, students in the massed condition outperformed students in the distributed condition, *t*(137) = -2.93, *p* = 0.004. At the longer retention interval, a slight and non-significant difference was found in the opposite direction between the massed (*M* = 1.79, *SE* = 0.34) and distributed condition (*M* = 2.34, *SE* = 0.40), *t*(137) = 1.05, *p* = 0.297.

These results showed that the predicted differential effects of massed vs. distributed learning at the short and long retention intervals were only partially supported. When students reread the text in a distributed fashion, no decrease in recall performance occurred from the short to the long retention interval. Nevertheless, the benefit of distributed rereading at the longer retention interval predicted in Hypothesis 1 did not occur.

### Comprehension Performance

We estimated a generalized mixed model with students and items as random effects (random intercepts) and learning condition (contrast coded: massed = -1, distributed = 1), retention interval (contrast coded: short = -1, long = 1), prior knowledge (*z*-standardized), and item type (contrast coded: CR = 1, MC = -1) and their interactions as predictors with fixed effects and comprehension performance as dependent variable (Table 1).

**Table 1:** Estimated coefficients, standard errors and *z*-values for the generalized linear mixed model with text comprehension as dependent variable.

	Estimated coefficients	*SE*	*z*
(Intercept)	–0.695	0.316	–2.202^∗^
Learning condition (LC)	–0.082	0.098	–0.839
Retention interval (RI)	0.305	0.098	3.100^∗∗∗^
Item type	–0.542	0.304	–1.782
Prior knowledge (PK)	0.477	0.111	4.309^∗∗∗^
Learning condition × retention interval	–0.192	0.098	–1.949^∗^
Learning condition × item type	–0.006	0.052	–0.115
Learning condition × prior knowledge	0.087	0.111	0.787
Retention interval × item type	–0.072	0.052	–1.383
Retention interval × prior knowledge	0.011	0.110	0.103
Prior knowledge × item type	0.174	0.060	2.953^∗∗^
Learning condition × retention interval × item type	0.029	0.052	0.556
Learning condition × retention interval × prior knowledge	–0.090	0.110	–0.810
Learning condition × prior knowledge × item type	0.151	0.060	2.587 ^∗∗^
Retention interval × prior knowledge × item type	–0.117	0.060	–1.992^∗^
Learning condition × retention interval × item type × prior knowledge	–0.011	0.060	–0.180


*Learning condition (contrast coded: distributed = 1, massed = –1). Retention interval (contrast coded: immediate = –1, delayed = 1). Item type (contrast coded: CR = 1, SC = –1). Prior Knowledge was included z-standardized. ^∗^*p* < 0.05, ^∗∗^ p < 0.01, and ^∗∗∗^*p* < 0.001 (one-tailed for directional hypotheses).*

Similar to the model for recall performance, retention interval (β = 0.30, *SE* = 0.10, *z* = 3.10, *p* < 0.001, one-tailed) and prior knowledge (β = 0.48, *SE* = 0.11, *z* = 4.31, *p* < 0.001) exerted main effects on comprehension performance. Participants performed better at the short retention interval (probability = 0.41, *SE* = 0.07) than at the long retention interval (probability = 0.28, *SE* = 0.07). A difference of one standard deviation in prior knowledge corresponded to a 11% difference in the probability to provide a correct response. The main effect of learning condition was not significant, β = -0.08, *SE* = 0.10, *z* = -0.84, *p* = 0.201, one-tailed. Performance in the massed condition (probability = 0.37, *SE* = 0.07) did not differ from performance in the distributed condition (probability = 0.33, *SE* = 0.08). However, the model revealed a significant interaction between learning condition and retention interval, β = -0.19, *SE* = 0.10, *z* = -1.95, *p* = 0.026, one-tailed (Figure 2B). Consistent with the findings from the recall performance analysis, students in the massed condition showed a decrease in the text comprehension performance from the short (probability = 0.47, *SE* = 0.08) to the long retention interval (probability = 0.26, *SE* = 0.07), *z* = -3.97, *p* < 0.001. In contrast, no significant decrease in text comprehension performance was found in the distributed condition from the short (probability = 0.35, *SE* = 0.08) to the long retention interval (probability = 0.30, *SE* = 0.08), *z* = -0.81, *p* = 0.420. At the short retention interval, the difference between massed and distributed condition was statistically significant, *z* = -2.16, *p* = 0.031, whereas the difference at the long retention interval was not significant, *z* = 0.75, *p* = 0.455.

**FIGURE 3:**
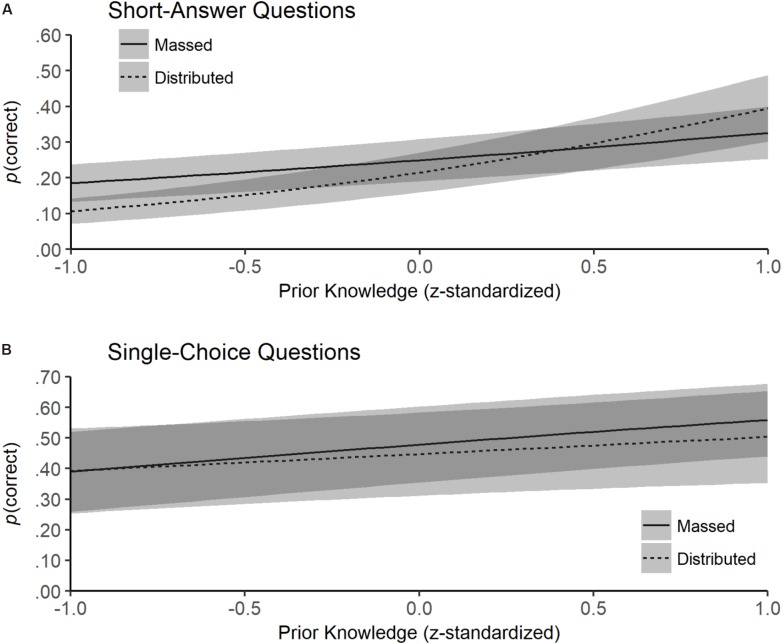
Back-transformed text comprehension performance (probability of correct answer) in **(A)** short-answer questions and **(B)** single-choice questions estimated as a function of prior knowledge and learning condition (massed vs. distributed). Shaded areas around each line represent standard errors.

Additionally, we found a significant three-way interaction between learning condition, prior knowledge, and item type, β = 0.15, *SE* = 0.06, *z* = 2.59, *p* = 0.010. The performance of students in the distributed condition was more strongly associated with prior knowledge than in the massed condition, but only with short-answer questions (Figure 3). To further interpret the interaction, we estimated and tested the effect of learning condition on the performance with short-answer questions for students with low prior knowledge (1 SD below the sample mean) and for students with high prior knowledge (1 SD above the sample mean; see Aiken and West, 1991, for a discussion on *post hoc* probing of continuous moderators). The analyses revealed that students with low prior knowledge showed lower comprehension performance in the distributed condition (probability = 0.10, *SE* = 0.03) than in the massed condition (probability = 0.18, *SE* = 0.05), *z* = -2.10, *p* = 0.036, whereas for students with high prior knowledge, no such difference was found between massed (probability = 0.33, *SE* = 0.07) and distributed conditions (probability = 0.39, *SE* = 0.09), *z* = 0.88, *p* = 0.380. The pattern of results for this type of question suggests that only students with lower prior knowledge were impeded by distributed rereading.

Summarizing the results of recall and text comprehension performance, Hypothesis 1, which stated that distributed rereading would have beneficial effects on learning in long-term retention, was not supported. In both learning outcomes, we found the interaction between learning condition and retention interval predicted in Hypothesis 1, but contrary to our assumptions, we found no benefit of distributed rereading at the longer retention interval. We found the decrease in both learning outcomes predicted in Hypothesis 2 but only in the massed condition. As predicted in Hypothesis 3, participants with higher prior knowledge showed better recall and text comprehension performance. Finally, our exploratory findings showed that participants with low prior knowledge seemed to be impeded by distributed rereading, whereas participants with higher prior knowledge benefitted equally from both reading conditions.

### Reading Behavior

**FIGURE 4:**
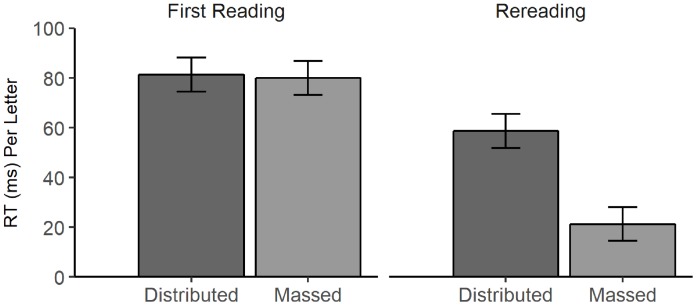
Estimated reading times per letter in the two learning conditions (massed vs. distributed) for the first reading and the rereading of the text. Error bars represent the standard error of the mean.

Reading times (first pass reading) were analyzed in a linear mixed model with sentences and students as random effects (random intercepts) and the fixed effects of learning condition (contrast coded: massed = -1, distributed = 1) and text presentation (contrast coded: first presentation = 1, second presentation = -1) and their interactions. It should be noted that the intraclass correlation for students missed the criterion value of 0.05, but it was included as random effect to achieve normal distribution of residuals. This model revealed a significant main effect of learning condition, β = 9.72, *SE* = 1.53, *t*(169) = 6.34, *p* < 0.001, indicating slower reading times in the distributed condition (*M* = 70.07, *SE* = 6.78) than in the massed condition (*M* = 50.62, *SE* = 6.72), and a main effect of text, β = 20.36, *SE* = 0.65, *t*(25062.99) = 31.26, *p* < 0.001. The second text presentation was read faster than the first, in the massed condition, *t*(25062.99) = 33.63, *p* < 0.001, and in the distributed condition, *t*(25062.99) = 11.72, *p* < 0.001. However, this difference was larger in the massed condition, as indicated by the significant interaction between learning condition and text presentation, β = -9.04, *SE* = 0.65, *t*(25062.99) = -13.88, *p* < 0.001 (Figure 4). Follow-up tests revealed that the reading times in the first presentation did not differ between the massed condition (*M* = 80.02, *SE* = 6.77) and the distributed condition (*M* = 81.38, *SE* = 6.85), *t*(235.51) = 0.41, *p* = 0.682. In contrast, in the second presentation, participants in the distributed condition (*M* = 58.75, *SE* = 6.85) read the text more slowly than participants in the massed condition (*M* = 21.23, *SE* = 6.77), *t*(235.51) = 11.27, *p* < 0.001.

In sum, the findings support Hypothesis 4 that distributed rereading would lead to longer reading times in the second text.

### Judgments of the Learning Process

For the perceived reading difficulty, predicted learning success analyses and on-task focus, we estimated linear models with the respective item(s) as dependent variable and learning condition (contrast coded: massed = -1, distributed = 1) as predictor.

#### Perceived Reading Difficulty

**FIGURE 5:**
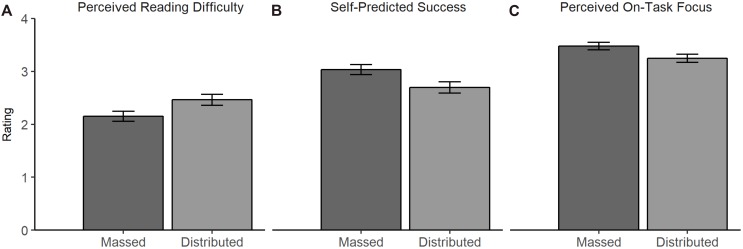
Estimated means in the judgments of the learning process for the massed and distributed condition. Part **(A)** shows the perceived reading difficulty, **(B)** the resulting self-predicted success, and **(C)** the perceived on-task focus during reading.

The effect of learning condition on perceived reading difficulty was not significant (Figure 5A); it failed to reach significance by a narrow margin, β = 0.11, *SE* = 0.07, *t*(169) = 1.65, *p* = 0.051, one-tailed, Δ*R^2^* = 0.02. Despite a descriptive difference between students in the distributed condition (*M* = 2.38, *SE* = 0.10) and students in the massed condition (*M* = 2.15, *SE* = 0.09) in the predicted direction, Hypothesis 5 was not supported.

#### Predicted Learning Success

Learning condition exerted an effect on predicted learning success, β = -0.16, *SE* = 0.07, *t*(169) = -2.46, *p* = 0.008, one-tailed, Δ*R^2^* = 0.03. In line with Hypothesis 6, students in the distributed condition (*M* = 2.71, *SE* = 0.10) predicted lower learning success than students in the massed condition (*M* = 3.04, *SE* = 0.09) (Figure 5B).

#### Perceived On-Task Focus

Learning condition also had an effect on the perceived on-task focus during learning, β = -0.13, *SE* = 0.05, *t*(511) = -2.70, *p* = 0.004, one-tailed, Δ*R^2^* = 0.01, but in the opposite direction than predicted in Hypothesis 7. Students reported higher on-task focus when rereading in a massed fashion (*M* = 3.49, *SE* = 0.07) compared to a distributed fashion (*M* = 3.23, *SE* = 0.07) (Figure 5C).

## Discussion

In this experiment, we investigated the effects of massed vs. distributed rereading on learning outcomes (recall and text comprehension performance) at two retention intervals, immediately after reading the text and 1 week later. We found a benefit for massed rereading at the short retention interval. At the longer retention interval, we found no difference between the learning conditions because of the lower forgetting rate in the distributed condition. In fact, the learning outcomes decreased between the retention intervals only in the massed condition, whereas students in the distributed condition showed no forgetting from the immediate to the delayed test of recall and comprehension performance. As a result, learning outcomes at the longer retention interval were on par for massed and distributed rereading but the distributed rereading condition did not show the expected advantage.

The main finding was that the effects of distributed rereading for secondary students depend on time of test, which parallels results found in earlier studies with college students. Distributed rereading seems to be detrimental when learning outcomes are assessed immediately, but it leads to a lower rate of forgetting that results in performance at least as good 1 week after learning. The difference in forgetting rates is in line with the previous studies by Rawson and Kintsch on distributed rereading (Rawson and Kintsch, 2005; Rawson, 2012). For example, Rawson (2012) found a decline of 49% for the massed (short-lag) condition, but only a decline of 3% for the distributed condition. By comparison, we found a decline of 50% in the massed condition and only 10% in the distributed condition. The difference in the decline of the distributed conditions might be explained by the length of the retention interval. Rawson’s (2012) delayed test was two days after learning, whereas the delayed test in the present study took place after 1 week.

The different patterns of learning outcomes at the two retention intervals raises the question of the underlying cognitive processes. Soderstrom and Bjork (2015) argue that short-term retention, assessed during learning or immediately after learning, rests on retrieval strength, i.e., on the currently accessible memory representations, whereas long-term retention relies on storage strength, which depends on the degree of interconnectedness of the learned information with other representations in long-term memory. For the latter, an unlimited capacity and no decrease over time is assumed (Bjork and Bjork, 1992). According to this approach, the goal of teaching and learning should be to increase storage strength and not retrieval strength. Importantly, a learning method which increases retrieval strength might even lead to lower increase in storage strength. To illustrate this assumption, Soderstrom and Bjork (2015) review several manipulations of learning situations which might have contrary effects on short- and long-term retention – and one of these might be distributed learning.

The pattern of effects for long- and short-term retention is also reminiscent of previous meta-analytic findings of the lag effect (Cepeda et al., 2006). As described above, the spacing effect does not depend on the retention interval, whereas the lag effect does. Moreover, Cepeda et al. (2006) reported from their meta-analysis spacing effects even for short retention intervals, and they found no evidence for the so-called Peterson paradox in which massed repetition is beneficial at short retention intervals. However, the distinction between spacing and lag effects depends on the definition of massed repetitions. For example, in Donovan and Radosevich’s (1999) definition, a massed repetition may be interrupted by items or time when necessary for the experimental design, whereas Cepeda et al. (2006) specified that massed learning means that the learning should not be interrupted at all. This evokes the question whether massed rereading is a massed repetition of learning materials as defined by Cepeda et al. (2006).

Massed rereading means that a text is read (e.g., 977 words in the present experiment), and immediately following the last sentence, the reader starts again with the first sentence. Thus, the repetition of each sentence is distributed by several sentences before the reader encounters the same sentence in the second reading. Consequently, Rawson (2012) used the term short-lag rereading instead of massed rereading. Although we agree with Rawson (2012, p. 870) that the term “massed is somewhat of a misnomer” as it is applied to rereading, we are not certain whether the term should be changed. Naming this condition short-lag would imply that a shorter lag is possible, but it is not with text materials. Additionally, when learning from text, the comprehension of the coherent text is essential, which depends not only on the information given within one sentence but also on its relation to other sentences in the text. Thus, the text should be considered as the unit of learning, and rereading always includes the text as a whole. Hence, the difference between massed rereading and other massed repetitions (e.g., single words) is clearly due to the nature of the materials. Moreover, the massed conditions employed in numerous studies in educational contexts do not fit the definition according to Cepeda et al. (2006) (Fishman et al., 1968; Harzem et al., 1976; Bloom and Shuell, 1981; Grote, 1995; Kornell, 2009; Paik and Ritter, 2016). In real-world learning settings, didactical strategies exclude pure massed repetitions, for example, when changing the repetition mode from reading to testing, as it was done in the study of Küpper-Tetzel et al. (2014). All of this implies that the pure spacing effect as defined by Cepeda et al. (2006) does not occur outside the laboratory. The research on distributed learning in real-world educational settings seems to investigate the lag effect rather than the spacing effect and thus might lead to a differential pattern regarding the learning outcomes at different times of test.

Several theories (e.g., the one-shot account of spacing, Delaney et al., 2010), use retrieval processes to explain the effects of distributed learning. This mechanism might be especially important for the explanation of the lag effect in which forgetting between the repetitions of learning materials is essential. Because of the inter-study interval, the last presentation of an item must be retrieved from memory, which is more difficult when the inter-study interval is longer. Furthermore, the more difficult the retrieval, the stronger the memory trace (Bjork, 1975). Generalizing these ideas to rereading, the information acquired during the first reading of a text has to be retrieved from memory when rereading the text. In a massed presentation of the text, information acquired during the first reading is easily retrieved, whereas in distributed presentation, the retrieval is more difficult. This might result in a stronger memory trace, which is more resistant to forgetting compared to massed rereading. This interpretation is well in line with our finding that distributed rereading prevented forgetting.

Further research might additionally address the question whether the retrievability of information acquired during the first reading plays a crucial role in beneficial effects of distributed rereading and contrast its effect on short- and long-term retention.

Bearing the assumption in mind, that distributed rereading is more related to the lag effect than to the spacing effect, the proportion of the inter-study and retention intervals might appear to not have been well chosen in the present study. According to Cepeda et al. (2008), the optimal inter-study interval for a 1-week retention interval would have been one or two days (20–40% of the retention interval), or the optimal retention interval for a 1-week inter-study interval would have been 18–35 days (note that these recommendations are based on experiments with simple verbal materials, not texts). In this experiment, we decided to use a retention interval which was as long as the inter-study interval. This was chosen for two different but related reasons. Most topics in school are taught on a weekly basis. Hence, testing the content of the previous lesson is often conducted 1 week later. For a more pragmatic reason, we also chose a schedule that fits well in the class learning schedule. Nevertheless, in further experiments, a longer retention interval and a better fit between retention interval and lag should be considered. Given the finding that distributed rereading changed forgetting from the short to the long retention interval, an effect of distributed rereading could emerge with a longer retention interval.

The findings from the prior knowledge analysis support the general assumption that students learn more from texts when their prior knowledge is already high (Schneider et al., 1989; Kintsch, 1998). For text comprehension performance assessed with short-answer questions, we also found a hint that the effects of distributed rereading depend on prior knowledge. Students with higher prior knowledge benefitted equally from distributed and massed rereading, whereas students with lower prior knowledge were hindered by the difficulties of distributed rereading. This finding is consistent with the idea that the learning difficulty introduced by distributed reading cannot be overcome by learners if the prior knowledge is too low. However, this relationship was not found in the free recall task and for single-choice questions.

We also found longer reading times in the second text presentation in the distributed condition compared to the massed condition. This pattern is comparable with the findings of Rawson (2012). In both experimental groups, the reading times during rereading were shorter than during the first reading. However, this decline was higher for the massed condition (74%) than for the distributed condition (24%) and both conditions declined to a greater extent compared to the rates reported by Rawson (2012), who found a decline of 14% for the distributed condition and 22% for the massed (short-lag) condition. School-aged students (at least in the age group that we looked at) might be even more vulnerable for rereading effects, especially when rereading takes place immediately. From this perspective, the extent that seventh-graders in the massed condition engaged in meaningful processing of the text during rereading is questionable. Apparently, though, at least some of the students engaged in meaningful processing at least to some extent. Otherwise, the superior performance of students in the massed condition at the short retention interval compared to students in the distributed condition would be difficult to explain. Nevertheless, given the results regarding the reading times, distributing the time of rereading might be even more essential in younger learners than in adults to prevent superficial processing of the text.

Students’ meta-cognitive judgments of the learning process might indicate that distributed rereading is perceived as more difficult than massed rereading. Consistent with our assumptions, students in the distributed condition predicted lower learning success. However, the descriptive difference between the conditions regarding the perceived difficulty showed a trend in the predicted direction but missed statistical significance. Furthermore, contrary to our initial assumption, students in the massed condition perceived higher on-task focus during reading. This is especially surprising considering the shorter rereading times in the massed condition. Maybe the longer session in the massed condition was perceived as more demanding and difficult, but the students confused this feeling with being on-task. Thus, distributed rereading might be perceived as more difficult, but this was not fully reflected by differences in the judgment of reading difficulty. Nevertheless, in sum, the results regarding the judgments of learning fit well with the assumption that distributed rereading is qualified as desirable difficulty.

Its informative results notwithstanding, this study suffers from certain limitations. As discussed above, maybe the biggest limitation (that is shared with other experiments on distributed rereading) is that we compared only two retention intervals and two learning intervals. Such a design provides only a snapshot of learning and may generate results that are not easy to interpret. In future research, it would be desirable to contrast several learning and retention intervals to get an insight into lag-effects in distributed rereading. However, an experiment based on such a complex design would not be easy to implement in a school setting. Further limitations are associated with the greatest advantage of our study, its implementation in the classroom. Of course, the real-world educational setting may lead to compromises regarding the control of potential distractors and interruptions of the individual learning process, which might have added some noise to our results (although systematic confounds are unlikely given the rigorous experimental design). Last but not least, the participants in this experiment read just two texts. The text topic was chosen carefully to match typical contents of the school curriculum and the texts were carefully designed to match typical expository texts for secondary school students. Nevertheless, the generalizability of results to other topics and texts is not entirely clear.

To conclude, this experiment was the first to replicate a central finding of distributed rereading with school-aged learners in a real-world learning setting: The effects of distributed rereading depend on the time of the test. The findings for meta-cognitive judgments highlight that learners perceive distributed rereading of text as difficult, and the findings for reading times suggest that the cognitive effort of readers is increased in distributed rereading. However, our results leave open the question of whether distributed rereading is also a desirable difficulty that should be promoted in school learning.

## Data Availability Statement

The dataset analyzed in this study can be found in the OSF Repository: https://osf.io/2sxu3/.

## Ethics Statement

For the reported study, no ethics approval was required per the guidelines of the University of Kassel or national guidelines. We conducted the study in line with the recommendations of the ethics committee of the University of Kassel and with approval of the “Ministry of Education and Cultural Affairs, Hesse, Germany (Hessisches Kultusministerium)” (cf. Education Act of Hesse, section 84). The parents of all participants gave written informed consent in accordance with the Declaration of Helsinki.

## Author Contributions

TR supervised the project, designed the research, and revised the manuscript. CG designed the research, organized the experimental conduction, analyzed and interpretated the data, and wrote the manuscript.

## Conflict of Interest Statement

The authors declare that the research was conducted in the absence of any commercial or financial relationships that could be construed as a potential conflict of interest.

## References

[B1] Aiken A. S. West S. G. (1991). *Multiple Regression: Testing and Interpreting Interactions.* Thousand Oaks, CA: Sage.

[B2] Alter A. L. Oppenheimer D. M. (2009). Uniting the tribes of fluency to form a metacognitive nation. *Personal. Soc. Psychol. Rev.* 13 219–235. 10.1177/1088868309341564 19638628

[B3] Amstad T. (1978). *Wie Verständlich Sind Unsere Zeitungen? [How understandable are our Newspapers?].* Zürich: Studenten-Schreib-Service.

[B4] Baayen R. H. Davidson D. J. Bates D. M. (2008). Mixed-effects modeling with crossed random effects for subjects and items. *J. Mem. Lang.* 59 390–412. 10.1016/j.jml.2007.12.005

[B5] Baddeley A. D. Longman D. J. A. (1978). The influence of length and frequency of training session on the rate of learning to type. *Ergonomics* 21 627–635. 10.1080/00140137808931764

[B6] Bates D. Mächler M. Bolker B. Walker S. (2015). Fitting linear mixed-effects models using lme4. *J. Stat. Softw.* 67 1–48. 10.18637/jss.v067.i01

[B7] Bjork E. L. Bjork R. (2011). “Making things hard on yourself, but in a good way: Creating desirable difficulties to enhance learning,” in *Psychology and the Real World: Essays Illustrating Fundamental Contributions to Society*, eds Gernsbacher M. A. Pew R. W. Hough L. M. Pomerantz J. R. (New York, NY: Worth Publishers), 56–64.

[B8] Bjork R. A. (1975). “Retrieval as a memory modifier: an interpretation of negative recency and related phenomena,” in *Information Processing and Cognition: The Loyola Symposium*, ed. Solso R. L. (Hillsdale, NJ: Lawrance Erlbaum Associates), 123–144.

[B9] Bjork R. A. (1994). “Memory and metamemory considerations in the training of human beings,” in *Metacognition: Knowing about Knowing*, eds Metcalfe J. Shimamura A. (Cambridge: MA: MIT Press),185–205.

[B10] Bjork R. A. Bjork E. L. (1992). “A new theory of disuse and an old theory of stimulus fluctuation,” in *From Learning Processes to Cognitive Processes: Essays in Honor of William K. Estes*, eds Healy A. Kosslyn S. Shiffrin R. (Hillsdale, NJ: Erlbaum), 35–67.

[B11] Bjork R. A. Dunlosky J. Kornell N. (2013). Self-regulated learning: beliefs, techniques, and illusions. *Annu. Rev. Psychol.* 64 417–444. 10.1146/annurev-psych-113011-143823 23020639

[B12] Bloom K. C. Shuell T. J. (1981). Effects of massed and distributed practice on the learning and retention of second-language vocabulary. *J. Educ. Res.* 74 245–248. 10.1080/00220671.1981.10885317

[B13] Callender A. A. McDaniel M. A. (2009). The limited benefits of rereading educational texts. *Contemp. Educ. Psychol.* 34 30–41. 10.1016/j.cedpsych.2008.07.001 26173288

[B14] Cepeda N. J. Pashler H. Vul E. Wixted J. T. Rohrer D. (2006). Distributed practice in verbal recall tasks: a review and quantitative synthesis. *Psychol. Bull.* 132 354–380. 10.1037/0033-2909.132.3.354 16719566

[B15] Cepeda N. J. Vul E. Rohrer D. Wixted J. T. Pashler H. (2008). Spacing effects in learning a temporal ridgeline of optimal retention. *Psychol. Sci.* 19 1095–1102. 10.1111/j.1467-9280.2008.02209.x 19076480

[B16] Delaney P. F. Verkoeijen P. P. J. L. Spirgel A. (2010). “Spacing and testing effects: a deeply critical, lengthy, and at times discursive review of the literature,” in *Psychology of Learning and Motivation: Advances in Research and Theory*, ed. Ross B. (San Diego, CA: Elsevier Academic Press), 63–147. 10.1016/S0079-7421(10)53003-2

[B17] Donovan J. J. Radosevich D. J. (1999). A meta-analytic review of the distribution of practice effect: now you see it, now you don’t. *J. Appl. Psychol.* 84 795–805. 10.1037/0021-9010.84.5.795

[B18] Fishman E. Keller L. Atkinson R. C. (1968). Massed versus distributed practice in computerized spelling drills. *J. Educ. Psychol.* 59 290–296. 10.1037/h0020055 5672245

[B19] Gagnon M. Cormier S. (2018). Retrieval practice and distributed practice: the case of French Canadian Students. *Can. J. Sch. Psychol.* 10.1177/0829573518773225

[B20] Gathercole S. E. Pickering S. J. Ambridge B. Wearing H. (2004). The structure of working memory from 4 to 15 years of age. *Dev. Psychol.* 40 177–190. 10.1037/0012-1649.40.2.177 14979759

[B21] Glenberg A. M. (1976). Monotonic and nonmonotonic lag effects in paired-associate and recognition memory paradigms. *J. Verbal Learn. Verbal Behav.* 15 1–16. 10.1016/S0022-5371(76)90002-5

[B22] Glover J. A. Corkill A. J. (1987). Influence of paraphrased repetitions on the spacing effect. *J. Educ. Psychol.* 79 198–199. 10.1037/0022-0663.79.2.198

[B23] Gluckman M. Vlach H. A. Sandhofer C. M. (2014). Spacing simultaneously promotes multiple forms of learning in children’s science curriculum: spacing, memory, and generalization. *Appl. Cogn. Psychol.* 28 266–273. 10.1002/acp.2997

[B24] Gordon K. (1925). Class results with spaced and unspaced memorizing. *J. Exp. Psychol.* 8 337–343. 10.1037/h0064623

[B25] Goossens N. A. M. C. Camp G. Verkoeijen P. P. J. L. Tabbers H. K. Bouwmeester S. Zwaan R. A. (2016). Distributed practice and retrieval practice in primary school vocabulary learning: a multi-classroom study. *Appl. Cog. Psychol.* 30 700–712. 10.1002/acp.3245

[B26] Greving C. E. Richter T. (2017). *The Effects of Distributed Rereading and Retention Interval in 7th Graders.* Available at: https://osf.io/7etq5/?view_only=29ff9b8df5a34e9c9ebb1d4ba530ea3b

[B27] Grote M. G. (1995). Distributed versus massed practice in high school physics. *Sch. Sci. Math.* 95 97–101. 10.1111/j.1949-8594.1995.tb15736.x

[B28] Harzem P. Lee I. Miles T. R. (1976). The effects of pictures on learning to read. *Br. J. Educ. Psychol.* 46 318–322. 10.1111/j.2044-8279.1976.tb02328.x

[B29] Inquisit 3 (2011). *[Computer Software].* Available at: http://www.millisecond.com

[B30] Kapler I. V. Weston T. Wiseheart M. (2015). Spacing in a simulated undergraduate classroom: long-term benefits for factual and higher-level learning. *Learn. Instr.* 36 38–45. 10.1016/j.learninstruc.2014.11.001

[B31] Karpicke J. D. Butler A. C. Roediger H. L. III (2009). Metacognitive strategies in student learning: do students practise retrieval when they study on their own? *Memory* 17 471–479. 10.1080/09658210802647009 19358016

[B32] Kintsch W. (1998). *Comprehension: A Paradigm for Cognition.* Cambridge, MA: Cambridge University Press.

[B33] Koriat A. (1997). Monitoring one’s own knowledge during study: a cue-utilization approach to judgments of learning. *J. Exp. Psychol.* 126 349–370. 10.1037/0096-3445.126.4.349 26443084

[B34] Kornell N. (2009). Optimising learning using flashcards: spacing is more effective than cramming. *Appl. Cogn. Psychol.* 23 1297–1317. 10.1002/acp.1537

[B35] Krug D. Davis T. B. Glover J. A. (1990). Massed versus distributed repeated reading: a case of forgetting helping recall? *J. Educ. Psychol.* 82 366–371. 10.1037/0022-0663.82.2.366

[B36] Küpper-Tetzel C. E. (2014). Strong effects on weak theoretical grounds: understanding the distributed practice effect. *Z. f. Psychol.* 222 71–81.

[B37] Küpper-Tetzel C. E. Erdfelder E. (2012). Encoding, maintenance, and retrieval processes in the lag effect: a multinomial processing tree analysis. *Memory* 20 37–47. 10.1080/09658211.2011.631550 22171809

[B38] Küpper-Tetzel C. E. Erdfelder E. Dickhäuser O. (2014). The lag effect in secondary school classrooms: enhancing students’ memory for vocabulary. *Instr. Sci.* 42 373–388. 10.1007/s11251-013-9285-2

[B39] Kuznetsova A. Brockhoff P. Christensen R. (2017). lmerTest package: tests in linear mixed effects models. *J. Stat. Softw.* 82 1–26. 10.18637/jss.v082.i13

[B40] Lenth R. V. (2016). Least-squares means: the R package lsmeans. *J. Stat. Softw.* 69 1–33. 10.18637/jss.v069.i01

[B41] Lipowsky F. Richter T. Borromeo-Ferri R. Ebersbach M. Hänze M. (2015). Wünschenswerte Erschwernisse beim Lernen. *Schulpädag. heute* 6 1–10.

[B42] McNamara D. S. Kintsch E. Songer N. B. Kintsch W. (1996). Are good texts always better? Interactions of text coherence, background knowledge, and levels of understanding in learning from text. *Cogn. Instr.* 14 1–43. 10.1207/s1532690xci1401_1

[B43] McNamara D. S. Kintsch W. (1996). Learning from texts: effects of prior knowledge and text coherence. *Discourse Proc.* 22 247–288. 10.1080/01638539609544975

[B44] Oberauer K. Süß H.-M. Schulze R. Wilhelm O. Wittmann W. W. (2000). Working memory capacity—facets of a cognitive ability construct. *Pers. Individ. Differ.* 29 1017–1045. 10.1016/S0191-8869(99)00251-2

[B45] Paik J. Ritter F. E. (2016). Evaluating a range of learning schedules: hybrid training schedules may be as good as or better than distributed practice for some tasks. *Ergonomics* 59 276–290. 10.1080/00140139.2015.1067332 26136052

[B46] Perfetti C. A. Landi N. Oakhill J. (2005). “The acquisition of reading comprehension skill,” in *The Science of Reading: A Handbook*, eds Snowling M. J. Hulme C. (Oxford: Blackwell), 227–247.

[B47] R Developmental Core Team (2018). *R: A Language and Environment for Statistical Computing.* Vienna: R Foundation for Statistical Computing.

[B48] Rawson K. A. (2012). Why do rereading lag effects depend on test delay? *J. Mem. Lang.* 66 870–884. 10.1016/j.jml.2012.03.004

[B49] Rawson K. A. Kintsch W. (2005). Rereading effects depend on time of test. *J. Educ. Psychol.* 97 70–80. 10.1037/0022-0663.97.1.70

[B50] Richter T. van Holt N. (2005). ELVES: Ein computergestütztes Diagnostikum zur Erfassung der Effizienz von Teilprozessen des Leseverstehens [ELVES: a computer-based test for measuring efficiency of processes involved in reading comprehension]. *Diagnostica* 51 169–182.

[B51] Schiff R. Vakil E. (2015). Age differences in cognitive skill learning, retention and transfer: the case of the Tower of Hanoi Puzzle. *Learn. Individ. Differ.* 39 164–171. 10.1016/j.lindif.2015.03.010

[B52] Schlagmüller M. Schneider W. (2007). *Würzburger Lesestrategie-Wissenstest für die Klassen 7-12 (WLST 7-12) [Würzburg Reading Strategy Knowledge Test for Grades 7-12].* Göttingen: Hogrefe.

[B53] Schneider W. Körkel J. Weinert F. E. (1989). Domain-specific knowledge and memory performance: a comparison of high- and low-aptitude children. *J. Educ. Psychol.* 81 306–312. 10.1037/0022-0663.81.3.306

[B54] Seabrook R. Brown G. D. A. Solity J. E. (2005). Distributed and massed practice: from laboratory to classroom. *Appl. Cogn. Psychol.* 19 107–122. 10.1002/acp.1066

[B55] Shrout P. E. Fleiss J. L. (1979). Intraclass correlations: uses in assessing rater reliability. *Psychol. Bull.* 86 420–428. 10.1037/0033-2909.86.2.420 18839484

[B56] Sobel H. S. Cepeda N. J. Kapler I. V. (2011). Spacing effects in real-world classroom vocabulary learning. *Appl. Cogn. Psychol.* 25 763–767. 10.1002/acp.1747

[B57] Soderstrom N. C. Bjork R. A. (2015). Learning versus performance. *Perspect. Psychol. Sci.* 10 176–199. 10.1177/1745691615569000 25910388

[B58] Son L. K. Simon D. A. (2012). Distributed learning: data, metacognition, and educational implications. *Educ. Psychol. Rev.* 24 379–399. 10.1007/s10648-012-9206-y

[B59] Thios S. J. D’Agostino P. R. (1976). Effects of repetition as a function of study-phase retrieval. *J. Verbal Learn. Verbal Behav.* 15 529–536. 10.1016/0022-5371(76)90047-5

[B60] Toppino T. C. Kasserman J. E. Mracek W. A. (1991). The effect of spacing repetitions on the recognition memory of young children and adults. *J. Exp. Child Psychol.* 51 123–138. 10.1016/0022-0965(91)90079-8 2010724

[B61] Verkoeijen P. P. J. L. Rikers R. M. J. P. Özsoy B. (2008). Distributed rereading can hurt the spacing effect in text memory. *Appl. Cogn. Psychol.* 22 685–695. 10.1002/acp.1388

[B62] Vlach H. A. (2014). The spacing effect in children’s generalization of knowledge: allowing children time to forget promotes their ability to learn. *Child Dev. Perspect.* 8 163–168. 10.1111/cdep.12079

[B63] Vlach H. A. Sandhofer C. M. (2012). Distributing learning over time: the spacing effect in children’s acquisition and generalization of science concepts: spacing and generalization. *Child Dev.* 83 1137–1144. 10.1111/j.1467-8624.2012.01781.x 22616822 PMC3399982

[B64] Vössing J. Stamov-Roßnagel C. Heinitz K. (2017). Text difficulty affects metacomprehension accuracy and knowledge test performance in text learning. *J. Comput. Assist. Learn.* 33 282–291. 10.1111/jcal.12179

